# Protocol Biopsies on de novo Renal-Transplants at 3 Months after Surgery: Impact on 5-Year Transplant Survival

**DOI:** 10.3390/jcm10163635

**Published:** 2021-08-17

**Authors:** Florian Terrec, Johan Noble, Hamza Naciri-Bennani, Paolo Malvezzi, Bénédicte Janbon, Camille Emprou, Diane Giovannini, Lionel Rostaing, Thomas Jouve

**Affiliations:** 1 Nephrology, Hemodialysis, Apheresis and Kidney Transplantation Department, University Hospital Grenoble, 38043 Grenoble, France; fterrec@chu-grenoble.fr (F.T.); jnoble@chu-grenoble.fr (J.N.); hnaciribennani@chu-grenoble.fr (H.N.-B.); pmalvezzi@chu-grenoble.fr (P.M.); bjanbon@chu-grenoble.fr (B.J.); tjouve@chu-grenoble.fr (T.J.); 2 Univ. Grenoble Alpes, 38043 Grenoble, France; cemprou@chu-grenoble.fr (C.E.); dgiovannini@chu-grenoble.fr (D.G.); 3 Pathology Department, University Hospital Grenoble, 38043 Grenoble, France

**Keywords:** kidney transplantation, protocol biopsies, kidney-allograft survival, death-censored graft survival, renal biopsy

## Abstract

Background: In many centers, a protocol kidney biopsy (PKB) is performed at 3 months post-transplantation (M3), without a demonstrated benefit on death-censored graft survival (DCGS). In this study, we compared DCGS between kidney transplant recipients undergoing a PKB or without such biopsy while accounting for the obvious indication bias. Methods: In this retrospective, single-center study conducted between 2007 and 2013, we compared DCGS with respect to the availability and features of a PKB. We built a propensity score (PS) to account for PKB indication likelihood and adjusted the DCGS analysis on PKB availability and the PS. Results: A total of 615 patients were included: 333 had a PKB, 282 did not. In bivariate Kaplan–Meier survival analysis, adjusting for the availability of a PKB and for the PS, a PKB was associated with a better 5-year DCGS independently of the PS (*p* < 0.001). Among the PKB+ patients, 87 recipients (26%) had IF/TA > 0. Patients with an IF/TA score of 3 had the worst survival. A total of 144 patients (44%) showed cv lesions. Patients with cv2 and cv3 lesions had the worst 5-year DCGS. Conclusions: A M3 PKB was associated with improved graft survival independently of potential confounders. These results could be explained by the early treatment of subclinical immunological events. It could be due to better management of the immunosuppressive regimen.

## 1. Introduction

Chronic kidney disease (CKD) is a major medical condition that affects approximately 600 million people worldwide [1]. Kidney transplantation (KTx) is the best treatment for end-stage renal disease (ESRD). Indeed, KTx increases life expectancy, diminishes morbidity, improves quality of life, and decreases health-related costs when compared to dialysis [2,3,4]. However, despite medical advances over the last decades in the field of transplantation, the long-term survival of transplants has not significantly improved [5,6].

Causes of transplant loss are diverse: antibody or cell-mediated rejection, recurrent or de novo glomerulopathy, polyomavirus (BKV)-associated nephropathy, a medical or surgical event, or the recipient’s death [7].

Transplant kidney biopsies are the diagnostic cornerstone of transplant dysfunction. Transplant kidney biopsies can often establish a diagnosis, evaluate the severity of the lesions, predict response to treatment, and have prognostic value.

Recently, several studies have suggested that early acute-rejection episodes and chronic changes to the allograft are often subclinical without a concomitant rise in serum creatinine or proteinuria [8,9,10,11]. Hence, performing a preemptive renal-allograft biopsy may help identify acute or chronic rejection. This may, therefore, potentially alter the outcome of a renal allograft that could be amenable to treatment by detecting subclinical rejection (SCR) [12,13,14,15]. Early recognition and treatment of SCR may improve long-term renal outcomes [16,17].

Moreover, protocol biopsies can track chronic histological changes in different compartments of the allograft and so provide a more detailed picture of the allograft’s health. Protocol biopsies can also reveal unsuspected findings and influence therapy. Other potentially reversible chronic diseases, such as chronic T-cell or antibody-mediated rejection, de novo glomerulopathy or recurrent disease, BKV nephropathy, interstitial fibrosis and tubular atrophy (IF/TA), and calcineurin inhibitor (CNI) nephrotoxicity, may be detected, thus allowing therapy to be modified to limit ongoing graft injury [18,19,20,21].

Despite the benefits provided by protocol biopsies, it is uncertain whether they improve long-term death-censored graft survival (DCGS). DCGS depends on many potential biases, especially in fragile recipients. In this study, we compared the 5-year DCGS of recipients that underwent a protocol biopsy at early post-transplantation, i.e., at 3 months (M3) compared to those with no biopsy.

## 2. Patients and Methods

### 2.1. Study Design

We retrospectively included all patients that received a kidney transplant between 1 January 2007 and 31 December 2013. Our program has implemented a protocol kidney-allograft biopsy at M3 since 2009. We divided our cohort into three groups. The first group (C2009/B+) were KT recipients that had a protocol biopsy at M3 and had received a transplant between 1 January 2009 and 31 December 2013. The second group (C2009/B−) included transplant recipients that had received a kidney during the same period but had not undergone a protocol M3 biopsy (due to, e.g., contra-indications, as detailed in the results). The third group (C2007) included transplant recipients that had received a transplant between 1 January 2007 and 31 December 2008, which was before the establishment of a systematic protocol biopsy was implemented in our center, and therefore had not undergone a systematic kidney biopsy.

The hospital’s electronic medical records were used to collect demographic data on the recipients and their donors. We collected clinical, pharmacological, biological, and available histological data at baseline and at M1, M3, M6, M12, M24, M36, M48, and M60. All subjects gave their informed consent for inclusion before they participated in the study. The study was conducted in accordance with the Declaration of Helsinki. All medical data were collected from our database [CNIL (French national committee for data protection) approval number 1987785v0]. 

Pre-transplantation data were donors’ and recipients’ ages, HLA mismatches, pre-KTx HLA sensitization, graft rank, BMI, hypertension, and the presence of pretransplant or de novo post-transplant diabetes in a recipient.

Data collected between KTx and M3 were the need for a transfusion during the first 3 months after KTx (RBC+), delayed graft function, estimated glomerular filtration rate (eGFR), and proteinuria. Data collected after M3 were patient and graft survival rates, eGFR, and proteinuria.

Delayed graft function was defined as any dialysis session during the first week post-KTx. Pretransplant HLA sensitization was defined as the presence of anti-HLA antibodies prior to KTx (as assessed by a Luminex assay using the Immucor ^®^ platform). Early post-KTx transfusion was defined as any red-blood-cell transfusion during the first 3 months after surgery. We used the Chronic Kidney Disease Epidemiology Collaboration equation (CKD-EPI) to estimate the glomerular filtration rate [22].

### 2.2. Kidney Biopsies

The biopsies were assessed according to the 2007 Banff classification [23]. All biopsies were read by the same pathologist. Biopsies with a class 2, 3, or 4 diagnoses, without proteinuria and with stable kidney function, were considered as subclinical rejections (SCR). Lesions were graded according to the same classification with a focus on: glomerulitis (g), peritubular capillaritis (ptc), interstitial infiltration (i), tubulitis (t), intimal arteritis (v), allograft glomerulopathy (cg), fibrosis endarteritis (cv), arteriolar hyalinosis (ah, aah), interstitial fibrosis (ci), and tubular atrophy (ct). C4d positivity was evaluated using immunofluorescence. A biopsy was considered adequate provided it contained at least 10 glomeruli and at least one medium vessel. A “limit” was considered if there were >6 glomeruli but **<** or = 10 and no medium-size vessel and was considered not adequate if there were less than 6 glomeruli.

The biopsies were performed during an out-patient stay in the nephrology unit. The procedure was performed under ultrasound guidance with a 16G automatic needle. The kidney-graft sample was processed for light microscopy by fixing in alcohol-formol-acetic acid (AFA) (fluid and embedding in paraffin). Specimens were stained with Masson’s trichrome, hematoxylin and eosin, periodic acid-Schiff (PAS) reagent, and silver staining. The biopsies were read by two pathologists (GD and CE) and were graded according to Banff classification. The biopsies were assessed according to the 2007 Banff classification [23].

No other systematic biopsy was performed in our center during the 2007–2014 period. Any other biopsy that might have been performed was therefore a for-cause biopsy.

### 2.3. Immunosuppressive Regimen

Every patient received an induction therapy of either basiliximab (20 mg on day (D)1 and D4) or anti-thymocyte globulins. Basiliximab was given to patients with low immunologic risk or that were Epstein–Barr virus (EBV) seronegative with an EBV seropositive donor. Anti-thymocyte globulins were given at different dosages depending on the immunological risk: 1 mg per kg per day for 5 days for recipients with a high immunologic risk (anti-HLA antibody(ies), whether DSA or not); 0.75 mg per kg per day for 5 days if there was early (day 4) steroid withdrawal; and 0.5 mg per kg per day for recipients with low immunological risk.

The maintenance immunosuppression therapy included steroids, tacrolimus, mycophenolate mofetil (MMF). Prednisone, given to patients with low immunological risk, was decreased to 10 mg per day on D30, then to 5 mg per day before being discontinued at M3 if this was deemed reasonable based on eGFR stability, proteinuria, and the M3-biopsy results. Prednisone was maintained for patients with a high immunologic risk for at least the first year post-transplantation, at 5 mg per day. Finally, prednisone was withdrawn at D4 for low-immunological-risk diabetic and/or obese patients. MMF was started at 1 g bid per day for the first 2 weeks, then decreased to 500 mg b.i.d. Tacrolimus was started on day 0 for recipients that received basiliximab and on day 3 for recipients that received anti-thymocyte globulins. Doses were adapted to trough levels, with a target of between 8 and 12 ng/mL over the first 3 months and then between 5 and 8 ng/mL. This immunosuppressive strategy was maintained throughout the follow-up, with a homogeneous policy at our center. Everolimus might have been introduced in place of MMF starting in 2017, which was not considered in this study as it at most marginally impacted the cohort discussed here.

Regarding the treatment of rejection episodes (whether subclinical or not), high-dose IV steroid pulses (500 mg each day for three days) were used, and oral steroids were then resumed at a daily dose of 10 mg. Subclinical borderline lesions were managed according to the attending physician.

### 2.4. Propensity Score

Despite a systematic kidney biopsy policy, not all patients transplanted after 2009 underwent a biopsy. We therefore defined a propensity score to predict the odds of having a biopsy that was based on our 2009–2013 cohort, based on baseline data. We adjusted a logistic regression model, accounting for the following baseline items: donor and recipient ages, donor type (living vs. deceased), delayed graft function, graft rank, early RBC transfusion, pretransplant HLA sensitization, and BMI. Using this propensity score, we predicted the probability of undergoing a systematic biopsy for every single patient, including patients from the 2007–2009 cohort (who did not undergo a systematic biopsy).

This propensity score was then used as a covariate in the following models to account for the potential indication bias (not undergoing a biopsy might be a confusion factor since cofactors influencing graft survival might influence the probability of undergoing a biopsy). Using a 0.75 threshold on the propensity score, we defined patients “likely to undergo a biopsy” (propensity score > 0.75) or “unlikely to undergo a biopsy” (propensity score ≤ 0.75). In other words, contrary to another widespread use of the propensity score, we did not match patients with a similar probability of undergoing a systematic biopsy but included the propensity score as a covariate of the survival analysis.

### 2.5. Endpoints

The primary endpoint was the 5-year death-censored graft survival (DCGS), with right-censorship beyond 5 years, adjusted for the presence of a systematic M3 biopsy and for the propensity score (with a 0.75 threshold).

The secondary endpoints were the 5-year eGFR, with or without a biopsy, while accounting for the propensity score (with a 0.75 threshold); the 5-year transplant survival rate, with or without biopsy while accounting for the propensity score (0.75 threshold); the 5-year transplant survival rate while accounting for a biopsy-guided steroid withdrawal, and Banff class 2, 3, and 4 biopsies at 5-year DCGS according to the combined ci + ct score and the 5-year DCGS according to the cv score.

### 2.6. Statistical Analyses

Kruskal–Wallis tests were used for multi-group comparisons of continuous covariates, while Fisher’s exact test was used for discrete covariates. The propensity score was built as a logistic regression model, adjusted for the aforementioned covariates, to predict the baseline probability of undergoing a biopsy. Kaplan–Meier survival curves were compared using the log-rank test. A Cox proportional hazard model was built for multivariate survival analysis, adjusting for the propensity score as one of the covariates. This propensity-adjusted survival analysis was performed to account for the indication bias of the systematic kidney biopsy. The accepted alpha risk was 5%. All statistical analyses were performed using the R statistical software.

## 3. Results

### 3.1. Recipients’ Characteristics

**Figure 1:**
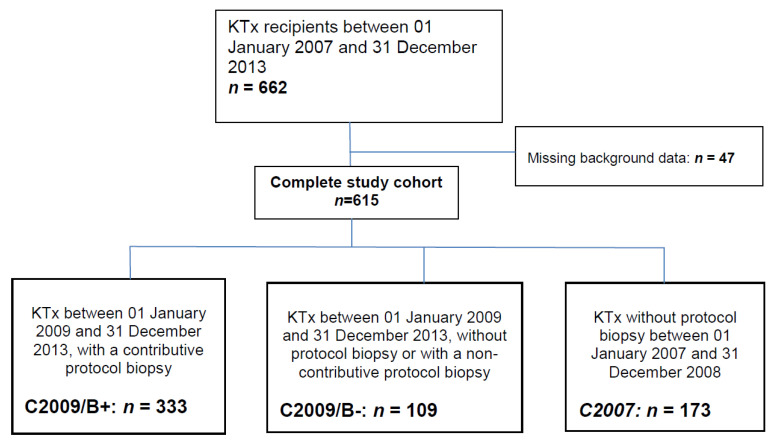
Study flowchart.

Between 1 January 2007 and 31 December 2013, 662 kidney transplantations were performed at Grenoble University Hospital. A completed data set (including HLA data, including biopsy data when performed) was available for 615 patients. Among these patients, an M3 protocol biopsy was performed in 367 recipients (group 2009–2013 biopsied recipients, C2009/B+). However, 34 of these biopsies did not contain enough material to make an interpretation: these patients were therefore considered as non-biopsy recipients (C2009/B−). Among recipients that did not undergo a systematic protocol biopsy, 173 had received a transplant between 1 January 2007 and 31 December 2008 (C2007), and 109 between 1 January 2009 and 31 December 2013 (C2009/B−). These three groups were included in the analyses (Figure 1). Reasons for not undergoing a systematic protocol biopsy in the C2009/B− cohort were ongoing anticoagulant therapy (*n* = 26, 23.8%), primary non-function (*n* = 22, 20.1%), earlier for-cause biopsy (*n* = 7, 6.4%), infectious event at the time of the planned systematic protocol biopsy (*n* = 7, 6.4%), intra-peritoneal location of the transplant (*n* = 6, 5.5%), coagulation disorders (*n* = 5, 4.6%), various and unknown (*n* = 36, 33%).

The baseline characteristics are summarized in Table 1. The overall mean of recipients’ age was 53.7 ± 14 years. C2007 and C2009/B+ patients were younger than C2009/B− (*p* < 0.001). The donor’s age also differed between groups (*p* < 0.001). BMI was similar in the three groups (*p* = 0.79), with an overall median of 24.6 kg/m^2^. Pretransplant HLA sensitization (i.e., having at least one positive HLA Luminex test before transplantation) was similar between the groups (*p* = 0.17), with an overall 26% of HLA sensitized patients. The dialysis vintage was similar in all three groups, with an overall median of 3.4 years (*p* = 0.2). An early transfusion (within 3 months post-transplantation) was required more often in the C2009/B− group: 43% vs. 26% for C2007 and 21% for C2009/B+ (*p* < 0.001).

The prevalence of pre-transplantation diabetes was similar in all three groups (*p* = 0.46), with an overall prevalence of 13%. In terms of pre-transplantation high blood pressure (*p* = 0.03), with an overall prevalence of 72%. The induction immunosuppressive therapy differed between the groups: 87% of C2009/B− recipients received ATG-based induction therapy vs. 93% in the C2007 and 82% in the C2009/B+ cohorts (*p* = 0.003).

### 3.2. Biopsy Characteristics: Banff Classification and Chronic Lesions

Among the 333 kidney biopsy samples, 217 (65%) were adequate (as defined by the Banff classification), 91(27%) were “limited”, and 25 (8%) were not adequate (lacking a sufficient number of glomeruli or an artery). Concerning the Banff classification categories, 212 (64%) biopsies were considered normal (class I), 88 (26%) had some level of IF/TA lesions (class V), 24 (7%) had subclinical immunological lesions (20 class III, and 4 class IV), and nine (3%) recipients had a class VI biopsy. Seven biopsies were class V associated with another lesion, either immunological (classes II, III, or IV, five recipients) or non-immunological (two recipients, class VI). Among the class VI biopsies, five patients showed a relapsing glomerulopathy (two cases of IgA, one of membranous nephropathy (MN), one had membranoproliferative glomerulonephritis (MPGN), one had sarcoidosis), two patients showed a de novo BKV nephropathy, one showed acute CNI toxicity, and one showed signs of pyelonephritis (Figure S1).

### 3.3. Chronic Lesions

**Figure 2:**
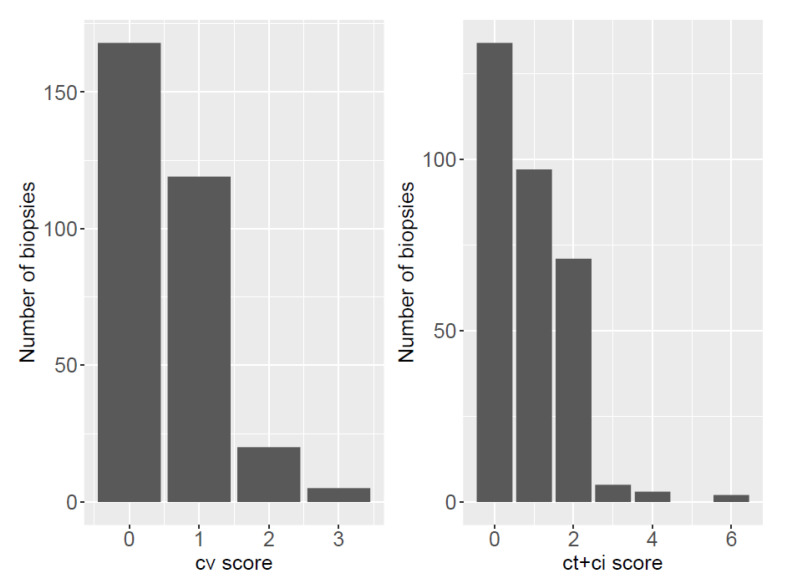
Barplot of chronic lesions (cv, IF/AT as the ci + ct score) among all available biopsies with a detailed Banff score (*n* = 312).

A total of 87 patients (26%) had some level of IF/TA, while there were 146 patients with cv lesions (44%). A total of 101 patients had ah lesions (30%), mostly of low grade (85 had an ah1 score). When considering chronic glomerular lesions, no biopsy showed signs of chronic allograft glomerulopathy (cg). Thirteen biopsies (4%) had mm lesions (Figure 2).

We investigated the ci + ct score: all patients with a biopsy ci + ct score ≤ 4 had the same 5-year DCGS as patients with a ci + ct score of 0, but patients with a score of 5 or 6 had a poorer survival (Supplementary Material (Figure S2)). As expected, a low number of patients had ci + ct > 3. Patients with cv2 and cv3 lesions had the worst 5-year DSGS (Figure S3). There was no statistical difference in terms of DCGS for ah and mm lesions.

### 3.4. Management of Subclinical Rejections

Subclinical rejections were heterogeneously managed after the M3 protocol biopsy. For borderline lesions (*n* = 20), all patients remained on oral steroids. In addition, seven of these patients with borderline lesions received high-dose pulses of steroids. Eleven had a control biopsy: 4/11 had persisting borderline lesions, and 2/11 evolved to T-cell-mediated rejection (TCMR) grade 1A. Regarding class IV biopsies, all patients responded adequately to high-dose pulses of steroids. There was, however, one graft loss at year 4 post-transplantation in a recipient with persisting TCMR lesions on control biopsies, 2 years after the M3 biopsy.

### 3.5. Propensity Score Coefficients

As described in Table 2, the variables most correlated with having a biopsy were the recipient’s age (−2.452, *p* = 0.014), graft rank (−3.47, *p* < 0.001), early RBC transfusion (−3.393, *p* < 0.001), and delayed graft function (−3.2, *p* = 0.001). To summarize, older recipients, patients with a second or a third graft, patients with a delayed graft function, and patients who received an early RBC transfusion (within the first 3 months) had the lowest probability of undergoing a systematic biopsy.

### 3.6. One-Year Biopsy Proven Acute-Rejection Rates

There were 3 cases (*n* = 1.7%) of BPAR during the 1st year post-transplantation in the C2007 group, and 19 cases (*n* = 4.3%) in the cumulated C2009/B− and C2009/B+ groups (*p* = 0.19). There was no association between early non-systematic allograft biopsy and BPAR at 1 year (*p* = 0.18).

### 3.7. Five-Year Death-Censored Graft Survival Rate with or without a Biopsy

Overall, the 5-year DCGS rate was excellent, with 90.6% survival. Regarding the 5-year DCGS, it was 73.4% in 2009/B- recipients, 95.5% in 2009/B+, and 91.2% in the C2007 cohort. These three groups were not compared due to the obvious

**Figure 3:**
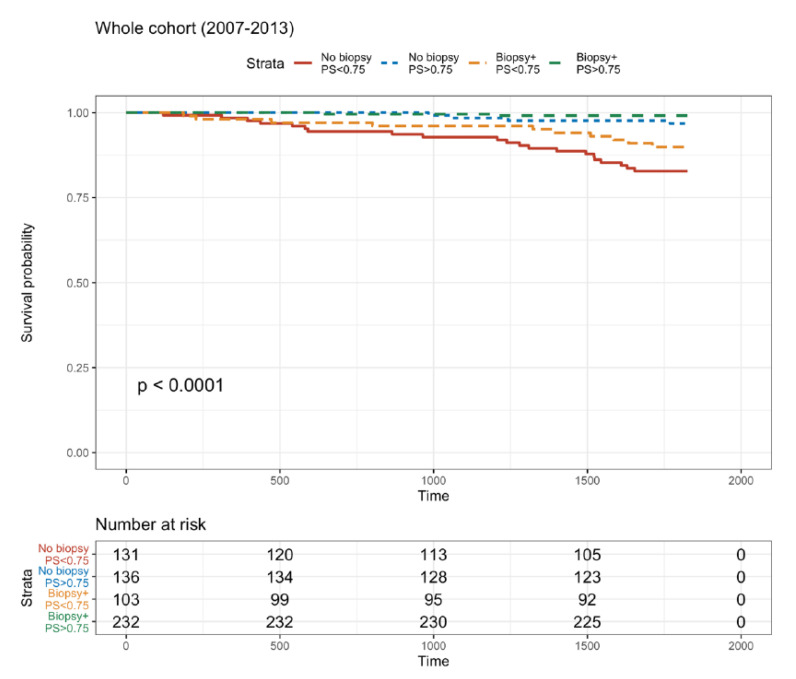
Five-year death–ensored graft survival (DCGS) for the whole cohort, with respect to systematic kidney biopsy at M3 and to the propensity score.

The primary endpoint was the 5-year DCGS with or without a biopsy while considering the PS. Four groups were defined based on the systematic biopsy (available results or not) and the propensity score (with a threshold of 0.75). There was a significant difference in survival between the four groups (log-rank test, *p* < 0.0001) (Figure 3). The recipients that underwent a systematic biopsy and had a high PS (>0.75) had the best survival (97%). The non-biopsied recipients with a low PS (<0.75) had the worst survival (83.9%). Interestingly, the group that did not have a systematic biopsy and a high PS and the group that had undergone a systematic biopsy but with a low PS had similar survival rates: i.e., 91.1% and 92.1%, respectively.

Similar results were obtained from the 2009–2013 cohort using the same four-group structure. Three different DGCS trajectories could be distinguished with the same grouping structure (Figure S4).

We also considered the effect of steroid withdrawal with respect to the availability of an M3 biopsy. Overall, 154/529 patients (29%) underwent a steroid withdrawal at M3 after kidney transplantation. Among these, only two patients lost their graft during follow-up, thus precluding any significant conclusion. Among the 84 patients with an early steroid withdrawal (from day 4 post-transplantation), performing a systematic biopsy was not predictive of DCGS (*p* = 0.336), while the propensity score remained a significant predictor (*p* = 0.012).

### 3.8. Five-Year Decline in eGFR According to Having or Not Having a Biopsy

**Figure 4:**
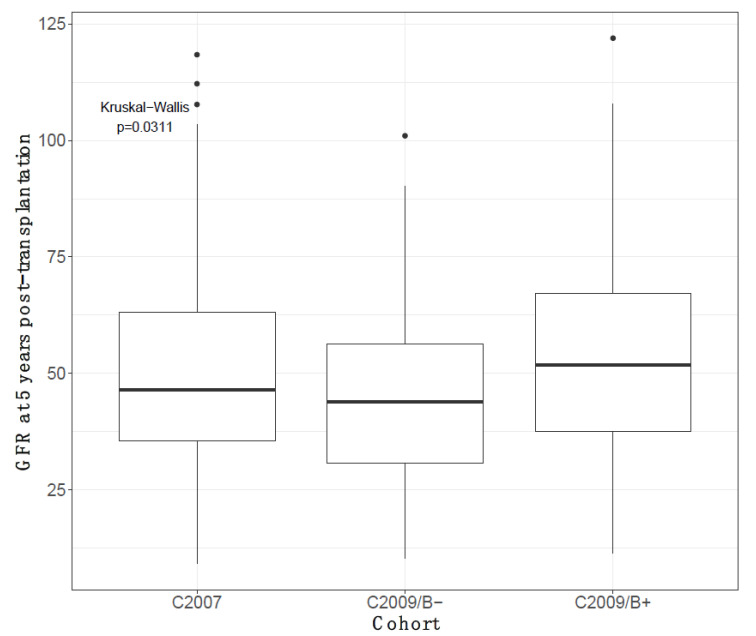
Five-year eGFR comparisons between the three different cohorts (CKD-EPI, mL/min/1.73 m^2^).

At 5-year post kidney transplantation, having undergone a biopsy was associated with a better kidney function (*p* = 0.031, Figure 4). Recipients from the C2009/B+ cohort had the best 5-year eGFR (51.9 mL/min/1.73 m^2^), followed by C2007 recipients (46.2 mL/min/1.73 m^2^), and C2009/B− recipients (44 mL/min/1.73 m^2^).

## 4. Discussion

In this study, we compared the 5-year DCGS according to whether kidney transplant recipients underwent a protocol M3 kidney biopsy or not while also accounting for baseline patients’ comorbidities through a propensity score. We show that performing an M3 protocol biopsy was associated with a better graft survival independently of potential confounding factors as assessed by the propensity score.

To the best of our knowledge, this is the first time that the presence of a systematic protocol biopsy has been evaluated in recipients treated with an immunosuppressive regimen based on tacrolimus.

In a randomized controlled study conducted in 1998 (that randomized for a systematic biopsy), the Winnipeg team showed that patients undergoing a systematic biopsy had lower short-term (2–3 months) and long-term (7–12 months) acute graft-rejection rates, a lower score for chronic interstitial and tubular lesions at M6, and, most importantly, better kidney function at M12 than patients that did not have a systematic biopsy [24]. More recently, in 2007, Kurtkoti et al. published a randomized controlled study on recipients that had living donors. A total of 52 recipients had an M3 systematic biopsy, and 50 did not. The two groups had the same rate of acute rejections during the first year post-transplantation, but kidney function was better in the systematic biopsy group, both at M6 (*p* = 0.0003) and M12 (*p* < 0.0001) [25]. In both these studies, patients received cyclosporine-based immunosuppression. Our results extend this finding to tacrolimus-based immunosuppression. It also gives a longer-term picture, over 5 years, and focused on the most stringent DCGS.

One could argue that protocol biopsies are associated with potential risks: indeed, they are not routinely performed. A recent survey of U.S. programs found that only 17% of centers routinely performed a protocol biopsy in all recipients, whereas other centers reserved a protocol biopsy for only higher-risk patients [26].

However, the fear of a biopsy causing a complication seems to be irrelevant. In a review by Wilczek [27], there was no graft loss when the biopsy was performed at least one month after kidney transplantation. In 2016, Morgan TA et al. showed that a systematic biopsy had a lower risk of complications than for-cause biopsies (2.7% vs. 0.33%, *p* < 0.001) [28]. The largest retrospective audit assessed major complications in 2127 adults’ kidney-allograft biopsies, and 1486 biopsies were assessed for minor complications in four major transplant centers in Europe. There were no deaths. One graft was lost under circumstances indicating that the loss could have been prevented. Three episodes of hemorrhage required direct intervention. Another prospective study from 2005 included 1171 protocol biopsies performed in 508 patients at 6, 12, and 26 weeks after transplantation. It also showed that the benefits of protocol biopsies outweighed the risks, with an acceptable complication rate [29].

In our analysis, by performing biopsies in patients with normal and stable graft function, we were able to detect subclinical rejections and unsuspected lesions (e.g., chronicity, CNI toxicity, recurrent or de novo glomerulopathy, BK virus nephropathy, and asymptomatic pyelonephritis). Evidence for these histological lesions was found in 33 patients (10%). We observed 24 (7%) cases of subclinical rejection. The rate of subclinical rejection was slightly lower than the rate observed in other studies that describe SCR on M3 systematic biopsies from recipients treated with tacrolimus: i.e., Gloor et al., in 2002, found 2.6% of s-TCMR and 11% of borderline lesions [30]; Moreso et al., in 2004, found 4.2 and 10% [31], respectively; and Mehta et al., in 2006, found 4 and 11%, respectively [32]. A multicenter prospective study in the current era of immunosuppression would be useful to identify the risk variables associated with SCR.

Confounding and interacting variables, such as cold-ischemia time and delayed graft function, may play significant roles in the occurrence of subclinical rejection.

The accurate management of these recipients by taking care of recurrent nephropathy, treating rejection, or avoiding steroid withdrawal could explain the beneficial effect of a systematic biopsy on graft survival.

As noted in the Results section, subclinical rejection was heterogeneously managed and treated after the initial biopsy. Most recipients had suitable outcomes. Indeed, among the 24 patients with SCR, only one recipient had TCMR on a control biopsy, and only one graft loss was recorded during follow-up.

Another explanation for the beneficial effect of a systematic biopsy could be better management of steroid withdrawal: biopsy-guided steroid withdrawal would be beneficial and allow steroids to be stopped in appropriate cases.

In this study, we failed to prove this effect because of the low number of patients actually weaned off steroids and the very low number of graft losses in our cohort. It is possible that undergoing a systematic biopsy reduced some of the undesirable withdrawal effects from steroids, thus increased graft survival. Several studies have shown the safety of steroid withdrawal after normal histological findings on the M3 PKB.

In 2003, Gotti et al. studied kidney transplant recipients receiving steroids, cyclosporine (CsA), and azathioprine. They were randomized to a per-protocol biopsy (*n* = 30) or no biopsy (*n* = 29) at 1–2 years post-transplantation. Steroids were withdrawn on the basis of histological findings. The effects of this withdrawal on drug-related complications, acute rejection, and graft function were assessed over a 3-year follow-up. After 3 years, patients underwent a second biopsy. Per-protocol biopsy histology revealed no lesions in only five of the patients where steroids had been discontinued. Reducing the drug regimen led to overall fewer side effects related to immunosuppression as compared to standard therapy or no biopsy. Steroids were safely discontinued with no acute rejection or graft loss [33].

In another study, Wehmeier C et al. analyzed 156 pretransplant DSA-negative renal transplant recipients receiving basiliximab as the induction therapy and maintenance immunosuppression of tacrolimus, mycophenolate, and steroids. The absence of rejection in surveillance biopsies at 3- or 6-months post-transplantation enabled steroid withdrawal. In the whole study population, successful steroid withdrawal was achieved in 73 of 156 patients (47%). Steroid withdrawal was initiated in 98 of 156 patients (63%) and was successful in 73 of 98 patients (74%). A subsequent clinical rejection event occurred in only one of 98 patients (1%), whereas 24 of 98 patients (24%) experienced a subclinical rejection. The authors concluded that successful steroid withdrawal is not predicted well by pre- and early post-transplant parameters and that guidance from a systematic surveillance biopsy was an interesting strategy [34]. These findings are important, given the fact that steroid withdrawal unguided by a biopsy may be a risk factor for early graft loss. Indeed, Haler et al. showed, in a retrospective cohort, that steroid withdrawal within 18 months after transplantation was associated with an increased rate of graft loss compared to steroid maintenance [35].

Recently, Domingo and al. performed a prospective, randomized controlled study conducted in low-immunological risk KT recipients, i.e., 105 patients were randomized, after a protocol biopsy at 3 months, to corticosteroid continuation (CSC, *n* = 52) or corticosteroid withdrawal (CSW, *n* = 53). Both groups received tacrolimus and MMF and had another protocol biopsy at 24 months. An increase in chronicity scores at 2-years post-transplantation was observed in low-immunological-risk KT recipients with initial no inflammation or subclinical inflammation. In addition, the authors showed that CSW may accelerate chronicity changes in patients with early subclinical inflammation but not for patients with no inflammation. Finally, a better cardiovascular profile may be achieved with this strategy [36].

There is currently no alternative to a protocol biopsy to track subclinical rejection or de novo and recurrent nephropathy. Moreover, systematic biopsies can show chronic lesions, which can help predict a future kidney function decline. In our study, we showed that recipients with the most deleterious lesions in terms of graft survival prognosis were cv > 1 and ci + ct > 4. In 2016, Cosio et al. showed that mild to moderate IF/TA, i-IF/TA, cg, and t lesions had a significant correlation with a poor DCGS compared to patients with normal to minor histological changes [37].

These findings can help us screen recipients that require further attention more efficiently. In the group of patients with the highest risk of graft loss due to chronic lesions, an immunosuppressive strategy with minimal reliance on calcineurin inhibitors could be useful. Such strategies include everolimus with low-dose tacrolimus [38] or tacrolimus withdrawal and a switch to belatacept [39].

In terms of 5-year death-censored graft survival, we can distinguish three different trajectories. The biopsied patients with a high PS had the best survival, non-biopsied patients with a low PS had the worst survival. In between these types of patients were the DCGS of biopsied recipients with a low PS and non-biopsied recipients with a high PS. These results emphasize our interest in systematic M3 allograft biopsies of all patients, and especially those with a high number of comorbidities.

One weakness of this study is its retrospective character, which we considered by adjusting the propensity score (of undergoing a protocol biopsy). This gave us the possibility of comparing survival rates between the biopsy and the no-biopsy groups. Using propensity score is known to be a suitable alternative method of analysis in non-randomized intervention trials. Nonetheless, the propensity score can only account for known confounding factors that were effectively measured. Equal distributions of unknown confounding factors can be achieved only in randomized controlled trials [40].

Another issue was that our work was monocentric: our results still have to be validated against an external validation cohort.

## 5. Conclusions

In this work, we suggest a beneficial impact of M3 protocol kidney-allograft biopsy on 5-year death-censored graft survival while also accounting for obvious biases through a propensity score. Interestingly, the beneficial impact was larger for the most comorbid recipients who routinely do not receive a biopsy. This suggests performing an M3 allograft biopsy every time there is no formal contraindication. Moreover, a protocol biopsy could inform decisions on potential steroid discontinuation. Finally, protocol biopsies may lead to adaptation to the immunosuppressive regimen in cases of chronic lesions, such as FIAT > 1 or cv > 1.

## Figures and Tables

**Table 1:** Recipients’ characteristics.

	C2007 ^1^ (*n* = 173)	C2009/B− ^2^ (*n* = 109)	C2009/B+ ^3^ (*n* = 333)	Total (*n* = 615)	*p*
Recipient age, year					0.001
Mean (SD)	50.9 (14.6)	57.1 (12.5)	51.8 (14.0)	52.5 (14.1)	
HBP history	138 (80%)	73 (67%)	236 (70%)	447 (72%)	0.031
Diabetes history	19 (11%)	16 (15%)	47 (14%)	82 (13%)	0.565
BMI, kg/m^2^					0.738
Mean (SD)	24.3 (4.1)	24.6 (4.2)	24.8 (4.9)	24.6 (4.6)	
PreTx HLA sensitization	40 (23%)	36 (33%)	83 (25%)	159 (26%)	0.149
Dialysis vintage, year					0.210
Mean (SD)	3.5 (5.1)	4.4 (5.7)	3.0 (3.3)	3.4 (4.4)	
Donor age, year					<0.001
Mean (SD)	47.7 (16.7)	56.9 (15.0)	52.0 (15.5)	51.6 (16.1)	
Donor type					0.017
Living	14 (8%)	9 (8%)	38 (11%)	61 (10%)	
BD	159 (92%)	98 (90%)	281 (84%)	538 (87%)	
DCD	0	2 (2%)	16 (5%)	18 (3%)	
Steroids on day 7 postTx					<0.001
Mean (SD)	6.5 (4.8)	8.4 (5.8)	9.3 (12.9)	8.3 (10.2)	
ATG induction	159 (92%)	95 (87%)	276 (82%)	530 (86%)	0.013
Early (first 3 months) blood transfusion	47 (27%)	48 (44%)	69 (21%)	164 (27%)	<0.001

^1^ C2007 mean of kidney transplant (KTx) recipients between 1 January 2007 and 31 December 2008, no protocol biopsy during this period ^2^ C2009/B− mean of KTx recipients between 1 January 2009 and 31 December 2013, with no protocol biopsies. ^3^ C2009/B+ mean of KTx recipients between 1 January 2009 and 31 December 2013, with a protocol biopsy, ATG: anti-thymocyte globulins, HBP: high blood pressure, BD = brain death, DCD = deceased from circulatory death.

**Table 2:** Propensity score details. The last column gives the statistical significance of the parameter in the propensity score.

	Estimate	Std. Error	z Value	Pr (>|z|)
(Intercept)	1.75	0.17	10.06	<0.001
Recipients’ age (years)	−0.036	0.015	−2.45	0.014
Donors’ age (years)	0.0011	0.012	0.087	0.93
Graft rank	−0.89	0.26	−3.47	<0.001
Early RBC transfusion (yes/no)	−0.88	0.26	−3.39	<0.001
Pre-immunization (yes/no)	0.12	0.30	0.41	0.68
Delayed graft function (yes/no)	−1.18	0.37	−3.19	0.001
BMI (kg/m^2^)	0.026	0.027	0.98	0.32

## Data Availability

Not applicable.

## Supplementary Materials

The following are available online at https://www.mdpi.com/article/10.3390/jcm10163635/s1, Figure S1: Repartition of Banff diagnoses in the Protocol Kidney Biopsy (PKB), Figure S2: Graft survival with respect to ci and ct lesions. The total number of available biopsies for this evaluation is *n* = 312 among the 333 patients with an available biopsy, due to a lack of precise ci and/or ct scores descriptions, Figure S3: Graft survival with respect to cv lesions. The total number of available biopsies for this evaluation is *n* = 312 among the 333 patients with an available biopsy, due to a lack of precise cv score descriptions, Figure S4: Graft survival for the 2009 cohort (biopsy: yes/no, PS > 0.75).

## Abbreviations

PKB	Protocol kidney biopsy
DCGS	Death-censored graft survival
PS	Propensity score
CKD	Chronic kidney disease
ESRD	End-stage renal disease
KTx	Kidney transplantation
BKV	BK polyomavirus
TKB	Transplant kidney biopsy
SCR	Subclinical rejection
IF/TA	Interstitial fibrosis and tubular atrophy
CNI	Calcineurin inhibitor
RBC+	Red-blood-cell transfusion
DGF	Delayed graft function
BMI	Body-mass index
CKD-EPI	Chronic Kidney Disease Epidemiology Collaboration equation
GFR	Glomerular filtration rate
CMV	Cytomegalovirus
EBV	Epstein–Barr virus
MMF	Mycophenolate mofetil
MN	Membranous nephropathy
MPGN	Membranoproliferative glomerulonephritis
s-ABMR	Subclinical antibody-mediated rejection
TCMR	T-cell-mediated rejection
