# Supplementary material for: Protocol Biopsies on de novo Renal-Transplants at 3 Months after Surgery: Impact on 5-Year Transplant Survival
Source: J Clin Med. 2021 Aug 17;10(16):3635. doi: 10.3390/jcm10163635 (PMC8397165; doi:10.3390/jcm10163635)

Supplementary Material

Figure S1: Repartition of Banff diagnoses in the Protocol Kidney Biopsy (PKB)

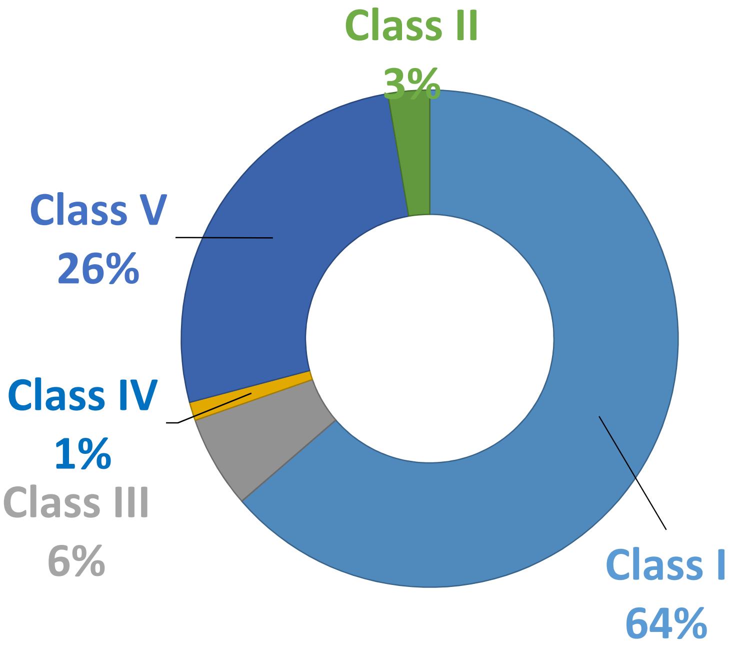

Figure S2: Graft survival with respect to ci and ct lesions. The total number of available biopsies for this evaluation is n=312 among the 333 patients with an available biopsy, due to a lack of precise ci and/or ct scores descriptions.

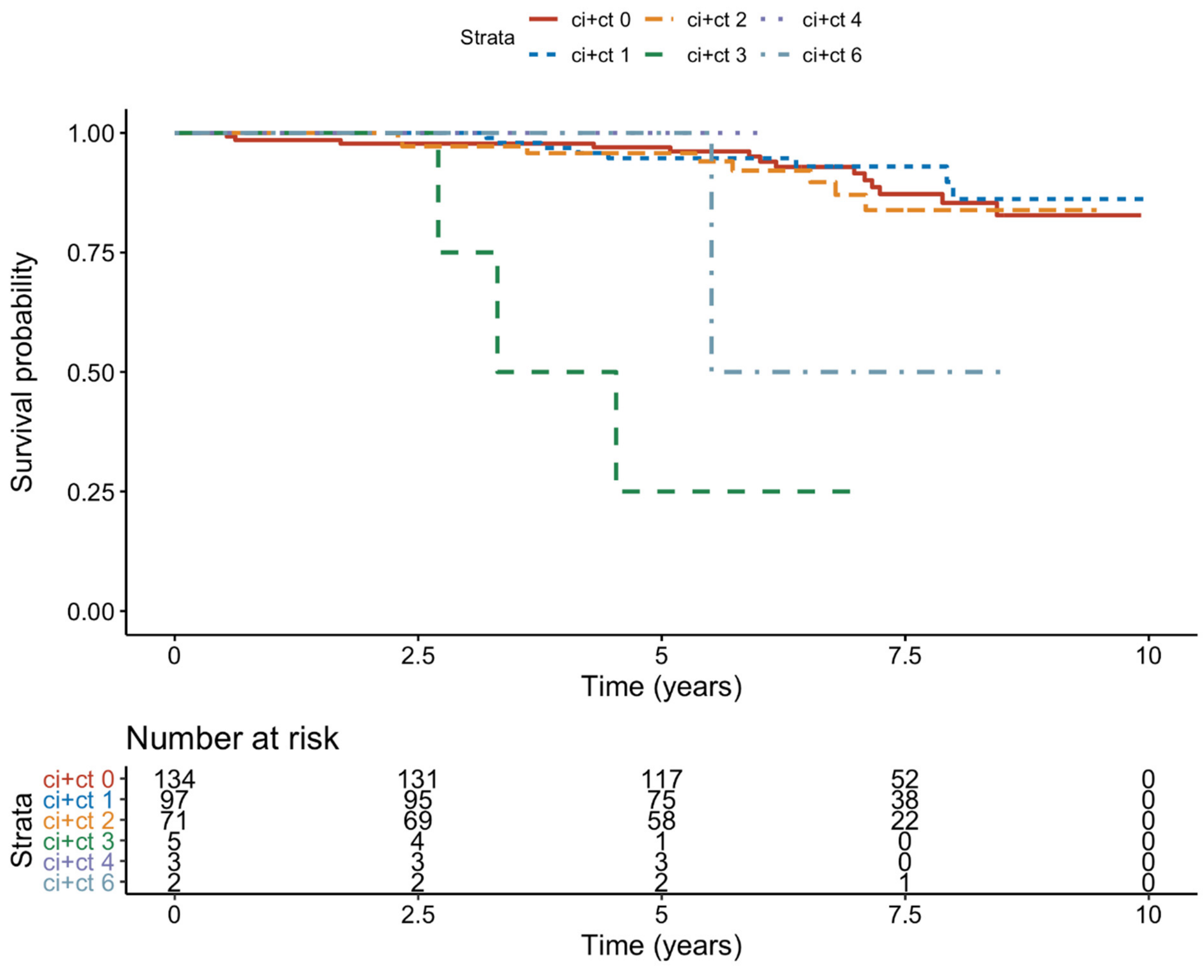

Figure S3: Graft survival with respect to cv lesions. The total number of available biopsies for this evaluation is n=312 among the 333 patients with an available biopsy, due to a lack of precise cv score descriptions.

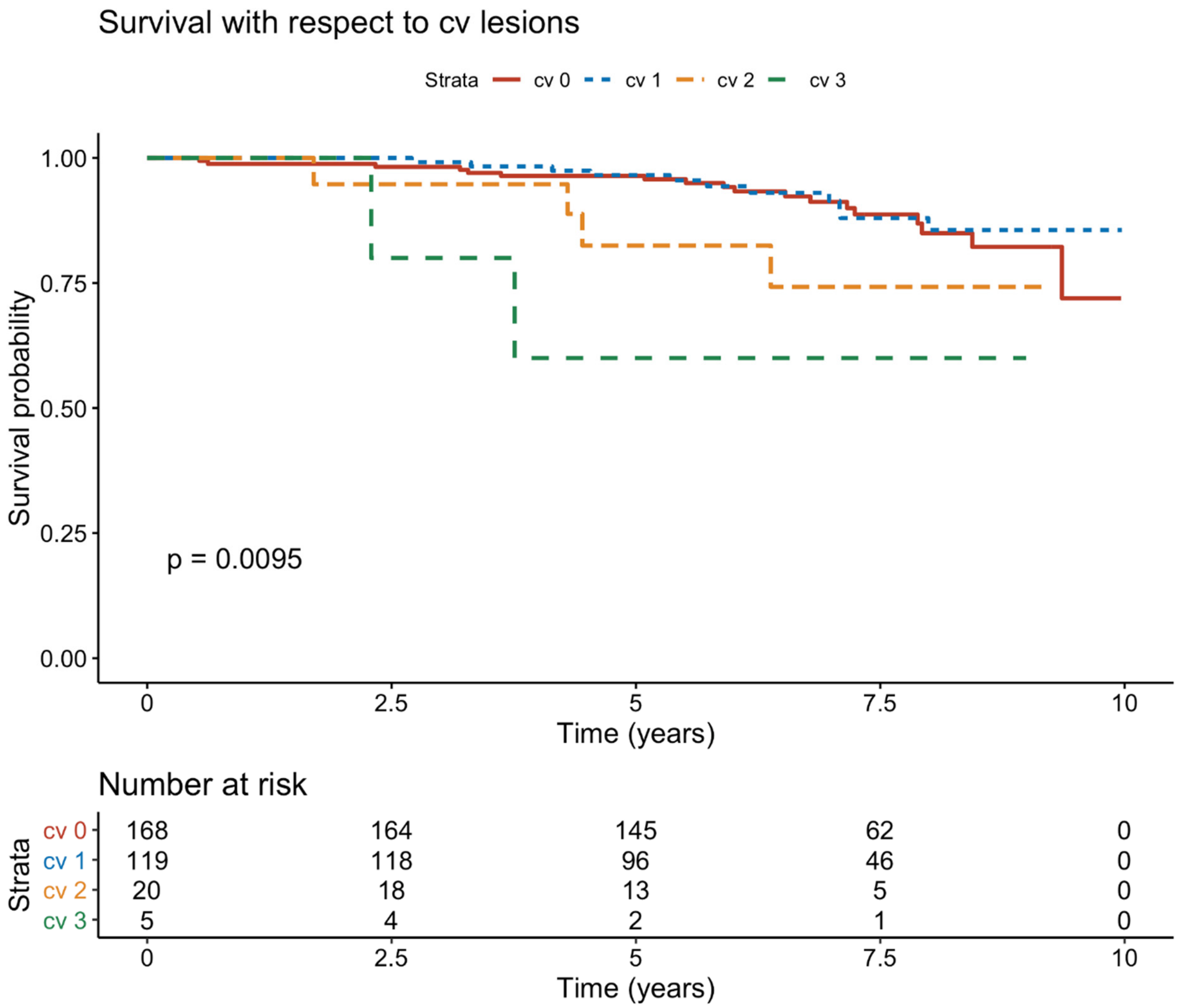

Figure S4: Graft survival for the 2009 cohort (biopsy: yes/no, PS >0.75)

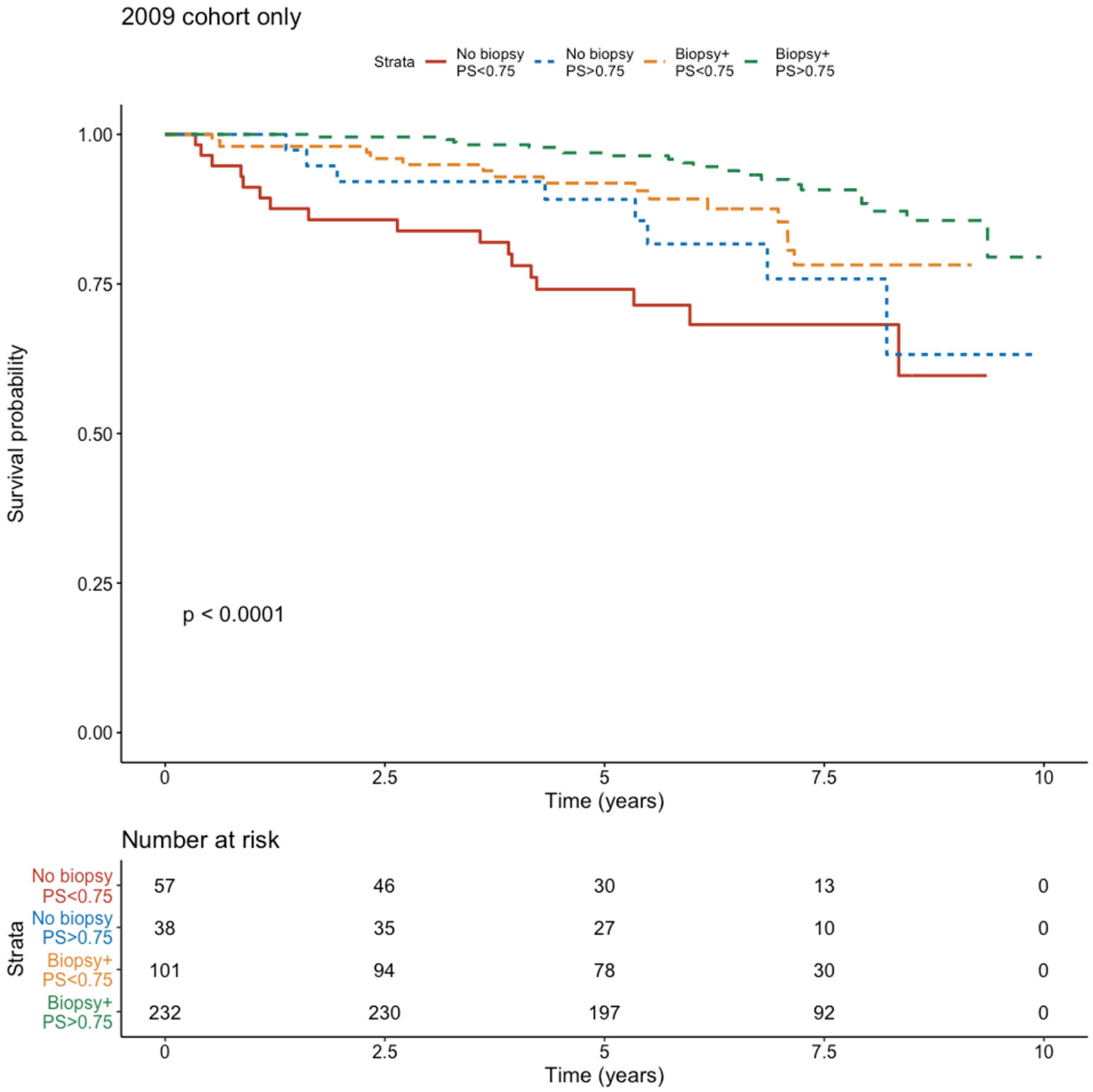

Supplement: Supplementary file 1 [file jcm-10-03635-s001.zip › jcm-1325080-supplementary.pdf]
